# Supplementary material for: Venomix: a simple bioinformatic pipeline for identifying and characterizing toxin gene candidates from transcriptomic data
Source: PeerJ. 2018 Jul 31;6:e5361. doi: 10.7717/peerj.5361 (PMC6074769; doi:10.7717/peerj.5361)
Supplement: Supplemental Information 6 [file peerj-06-5361-s006.gz › FinalOutput_1E-6/Kunitz-type_serine_protease_inhibitor_BmKTT-3_1/finaltree.pdf]

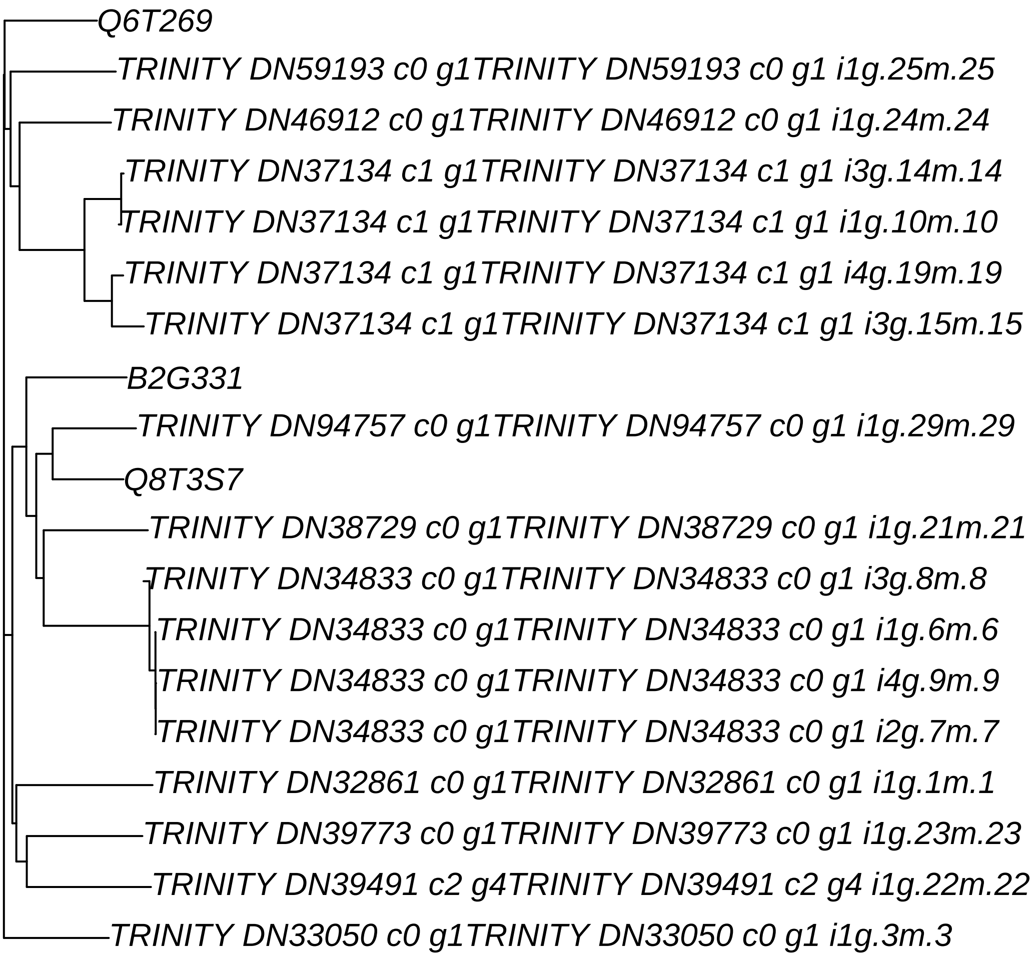
Q6T269
TRINITY DN59193 c0 g1TRINITY DN59193 c0 g1 i1g.25m.25
TRINITY DN46912 c0 g1TRINITY DN46912 c0 g1 i1g.24m.24
TRINITY DN37134 c1 g1TRINITY DN37134 c1 g1 i3g.14m.14
TRINITY DN37134 c1 g1TRINITY DN37134 c1 g1 i1g.10m.10
TRINITY DN37134 c1 g1TRINITY DN37134 c1 g1 i4g.19m.19
TRINITY DN37134 c1 g1TRINITY DN37134 c1 g1 i3g.15m.15
B2G331
TRINITY DN94757 c0 g1TRINITY DN94757 c0 g1 i1g.29m.29
Q8T3S7
TRINITY DN38729 c0 g1TRINITY DN38729 c0 g1 i1g.21m.21
TRINITY DN34833 c0 g1TRINITY DN34833 c0 g1 i3g.8m.8
TRINITY DN34833 c0 g1TRINITY DN34833 c0 g1 i1g.6m.6
TRINITY DN34833 c0 g1TRINITY DN34833 c0 g1 i4g.9m.9
TRINITY DN34833 c0 g1TRINITY DN34833 c0 g1 i2g.7m.7
TRINITY DN32861 c0 g1TRINITY DN32861 c0 g1 i1g.1m.1
TRINITY DN39773 c0 g1TRINITY DN39773 c0 g1 i1g.23m.23
TRINITY DN39491 c2 g4TRINITY DN39491 c2 g4 i1g.22m.22
TRINITY DN33050 c0 g1TRINITY DN33050 c0 g1 i1g.3m.3
